# Modeling Trait Anxiety: From Computational Processes to Personality

**DOI:** 10.3389/fpsyt.2017.00001

**Published:** 2017-01-23

**Authors:** James G. Raymond, J. Douglas Steele, Peggy Seriès

**Affiliations:** ^1^Institute for Adaptive and Neural Computation, University of Edinburgh, Edinburgh, UK; ^2^School of Medicine (Neuroscience), Ninewells Hospital and Medical School, University of Dundee, Dundee, UK

**Keywords:** anxiety, trait anxiety, anxiety disorders, computational modeling, associative learning, avoidance, attentional control

## Abstract

Computational methods are increasingly being applied to the study of psychiatric disorders. Often, this involves fitting models to the behavior of individuals with subclinical character traits that are known vulnerability factors for the development of psychiatric conditions. Anxiety disorders can be examined with reference to the behavior of individuals high in “trait” anxiety, which is a known vulnerability factor for the development of anxiety and mood disorders. However, it is not clear how this self-report measure relates to neural and behavioral processes captured by computational models. This paper reviews emerging computational approaches to the study of trait anxiety, specifying how interacting processes susceptible to analysis using computational models could drive a tendency to experience frequent anxious states and promote vulnerability to the development of clinical disorders. Existing computational studies are described in the light of this perspective and appropriate targets for future studies are discussed.

## Introduction

In psychological experiments, computational models can be used to capture individual differences in behavior and neural activity across a variety of contexts (1–3). For example, measured differences may be related to an underlying neurological condition (4–6). Research applying computational modeling to behaviors and neural activity associated with mental illness has enjoyed considerable growth in recent years and is collectively known as computational psychiatry (7–11). By drawing on theories of decision-making (12, 13), reinforcement learning (14), and Bayesian inference (12, 15–17), researchers have begun to explore the processes underlying psychiatric symptoms (18), classify individuals along symptom dimensions (19), and formulate theories about disease mechanisms in conditions such as depression [e.g., Ref. (20)], obsessive–compulsive disorder [e.g., Ref. (21)], autism [e.g., Ref. (22)], and schizophrenia [e.g., Ref. (23, 24)]. This work demonstrates considerable potential for computational methods to describe and explain behavioral differences pertaining to psychiatric illness.

Comparatively, little computational work has been carried out in relation to anxiety disorders, which have been described as the most common of all mental health problems (25, 26) and are highly costly for societies (27, 28). The current version of the Diagnostic and Statistical Manual of Mental Disorders (DSM-5) describes several anxiety disorders, including specific phobia, panic disorder, agoraphobia, social anxiety, and generalized anxiety disorder (GAD), all of which involve “anticipation of future threat” (29) and “tend to be highly comorbid with each other” (29). Anxiety is also featured in the National Institute of Mental Health Research Domain Criteria within the domain of Negative Valence Systems, where it is conceived as encompassing “responses to potential harm” (30). Continuing uncertainty about the best way to classify anxiety disorders (31–33) and advocacy for a dimensional perspective (34) has encouraged a focus on subclinical “trait” anxiety in work seeking to explore mechanisms underlying individual differences in response to diffuse threat (35).

Derived from self-report questionnaires, trait anxiety is a measure of the frequency with which symptoms of anxiety are experienced by an individual (36), or how characteristic they are of an individual in general (37). It is distinct from “state anxiety,” which is a measure of the intensity of anxiety experienced on comparatively short timescales (36, 38, 39). Although elevated levels of trait anxiety are a risk factor for the development of clinical anxiety disorders (40), it remains unclear how this risk is conferred. As a result, researchers hoping to bring the precision of computational methods to bear on the topic have no clear guide about which mechanisms to target and how they are involved in state, trait, and pathological anxiety.

This review draws on evidence from neuroscience, cognitive psychology, and existing computational studies to provide an integrated computational perspective on trait anxiety. Consistent with a network view of personality traits (41), its primary hypothesis is that the trait vulnerability consists in altered learning, rooted in biological differences, which leads over time to a characteristic profile of biases and behaviors that make anxious states more likely. In parallel, frequent anxious states calibrate the brain to anticipate a hostile environment, increasing the risk that anxiety will come to dominate an individual’s behavior [for a description of relevant biological mechanisms, see Ref. (42)]. By explicitly decomposing trait anxiety into subcomponents, this perspective provides starting points for future computational studies of anxiety-related biases in non-clinical populations.

There follows a brief introduction to methods commonly used in computational psychiatry, which may be unfamiliar to some readers. Subsequently, the relationships between anxious states, trait anxiety, and anxiety disorders are discussed, along with their relationships to self-reported trait anxiety scores that are often used as regressors in behavioral analysis. A description of neuroscientific background material is then used to motivate the decomposition of trait anxiety into biological risk factors, a resultant primary learning bias, and associated behavioral preferences. Existing computational studies that evaluate behaviors and biases associated with trait anxiety are described in detail and their findings situated within this schema. Finally, questions for future study are suggested.

## Computational Background

This review makes reference to four types of computational model: reinforcement learning models, models of decision processes, Bayesian models, and network models. Each model type has already been applied to the study of anxiety in one form or another—and detailed accounts of exemplar studies will be provided in the section on “Existing Computational Studies of Trait Anxiety.” The current section provides brief introductions along with references to relevant tutorial material and intuitive suggestions about how these modeling approaches could be used to capture individual differences associated with pathological anxiety. This is intended to assist the reader in understanding the computationally informed conceptualization of trait anxiety introduced in the sections on “State Anxiety, Trait Anxiety, and Anxiety Disorders” and “Trait Anxiety: Targets for Computational Studies” and the more in-depth discussion of computational studies and open questions in the sections on “Existing Computational Studies of Trait Anxiety” and “Outstanding Computational Questions.”

Computational models are precise descriptions of underlying processes thought to generate key aspects of observed behavior. Models force scientists to make explicit any assumptions about these processes by explicitly encoding them in the form of mathematical equations and computer programs. The relative power of different models to explain a given data set can be compared using model selection procedures (43, 44), which provide a principled way to decide which model (and, therefore, which set of assumptions) gives the best description of the underlying generative process. Furthermore, most models feature one or more parameters that can be adjusted to alter model performance. The most likely parameters to have produced experimental data can be found using well-established statistical methods and between-groups differences in fitted parameters can capture potential reasons for observed differences in behavior (unlike, say, a difference in reaction times, which might show that one group is faster than another without explicitly connecting this difference to any underlying generative process).

One popular source of models used to analyze the behavior of clinical populations (14) is the theory of reinforcement learning, which describes how agents adapt their behavior in the light of experience to maximize rewards and minimize punishments (45). Reinforcement learning models assign values to states (e.g., stages of an experiment) and actions (e.g., pressing a button) by ascribing higher values to states that feature rewards or actions that lead to better outcomes (e.g., for a biological organism, access to food, or avoidance of a predator). Actions are typically chosen using probabilistic action selection rules that bias choices toward high-valued actions; values, in turn, are continually revised on the basis of discrepancies between expected and actual outcomes, which are known as prediction errors. Two parameters are essential to this process: the “learning rate”—that determines how rapidly prediction errors alter value estimates and the “temperature”—that determines the degree of randomness in action choices. For high temperature settings, action selection will be exploratory so that new or low-valued actions continue to be sampled; at lower temperatures, action selection will be “greedy,” repeatedly exploiting previously successful actions.

A straightforward hypothesis concerning reinforcement learning in anxiety disorders is that patients are more likely than controls to repeat actions that previously allowed them to avoid an unpleasant outcome. Given a suitable experimental design, this tendency might correspond to a lower fitted temperature parameter in a reinforcement learning model, which increases the probability that reinforced actions are repeated. Since key quantities in reinforcement learning models have well-established neural correlates (46–49), measuring them in neuroimaging experiments could be an effective way to detect anxiety-related differences in neural activation [for a study addressing state anxiety, see Ref. (50)].

Sequential sampling models (51) describe how a decision is made to select one option among a finite number of alternatives on timescales usually less than 2–3 s. Decisions susceptible to analysis with such models are made rapidly and typically involve just two alternatives (52). A widely used subtype of sequential sampling model, the drift-diffusion model (DDM) (53, 54), models a decision as a particle moving randomly toward a boundary. DDMs combine information from response time distributions for correct and incorrect responses over many trials to calculate “drift rate” and “decision threshold” parameters that, respectively, express (i) how quickly the particle moves toward the boundary and (ii) how far it has to travel before a decision is made [for further information on derivation of these parameters, including model implementation, see Ref. (55–59)]. Since many threat-related biases observed among anxious individuals operate on timescales under 3 s [reviewed in Ref. (60)], DDMs ought to be able to capture their effects. Indeed, these models have already been used to examine cognitive biases relating to threat processing and classification among subjects with high levels of self-reported trait anxiety [see Existing Computational Studies of Trait Anxiety; also Ref. (61–64)].

Models of Bayesian inference describe how the probability associated with a hypothesis can be updated in the light of new evidence, such as experimental data. Crucially, new information is integrated with prior expectations in order to update beliefs about the world (65–68). Operating at a higher level of abstraction than either reinforcement learning or DDMs [both of which can be encompassed within a Bayesian framework; see Ref. (69, 70)], these models can be used to infer how subjects represent dynamic aspects of environmental states or which preferences they bring to bear on a particular problem. For example, a recent paper made use of a Bayesian model to infer how participants’ estimates of environmental volatility varied with self-reported levels of trait anxiety (71). Bayesian Decision Theory (BDT) (12, 69) can be used to express individual differences in deliberative, or prospective, decision-making that depend on individual preferences expressed in the form of utility functions. This too has clear relevance to the behavior of individuals with anxiety disorders, who are predicted to exhibit a number of distinctive preferences, including increased expected utilities of avoidance and threat detection. These and other preferences can be inferred from behavioral data collected using appropriate models and experimental designs [see, for example, Ref. (72)].

As these examples show, computational modeling is not only relevant to the study of anxiety, but has considerable advantages compared to more traditional methods of behavioral analysis that rely on statistical tests alone. Specifically, modeling can detect behavioral effects too subtle to be revealed by traditional methods (61, 72), isolate neural activity that tracks variables of interest (50), and provide theoretical explanations for variability in behavioral performance (71). These examples are described in more detail in the section on “Existing Computational Studies of Trait Anxiety.” A further type of modeling—network analysis—may offer an additional advantage: the capacity to understand and describe how different symptoms of anxiety disorders, or anxious behavior in general, interact and reinforce one another.

Network analysis is a way of representing and understanding interactions between subcomponents of complex systems like biological cells or social networks (73). As in the case of other computational approaches, the application of network analysis to the study of psychiatric symptoms is a relatively recent development (74–76). It was originally motivated by work on the theory of psychological measurement (77) and diagnostic systems (78), which described problems with a view of psychiatric disorders (78) or personality dimensions (41) that sees them as arising from a single hidden factor. According to this work, psychiatric illnesses are entirely constituted by characteristic sets of causally interacting symptoms—and not, like many physical illnesses, dependent on a single hidden variable that causes all the symptoms independently [(76, 78); see also Ref. (79)]. This idea has been used to explain patterns of comorbidity (74), transitions between health and disease (80), and vulnerability to mental illness (81). Network analysis in such studies generally proceeds by analyzing correlations between self-reported symptoms that suggest how they might be related—for example, that subgroups of symptoms within a disorder tend to cluster together (82) or that certain stressful life events are more likely to cause some symptoms of depression than others (83).

In relation to anxiety disorders, the same approach has been applied to self-report and experimental measures of anxiety from individuals with a diagnosis of social anxiety disorder (SAD) (84), facilitating analysis of correlations between these measures. The combination of network analysis methods with experimental measurement of attentional factors, which have an established albeit complex relationship with anxiety symptoms (85), demonstrates how network methods may be fruitfully combined with other experimental techniques. Their further combination with precise quantification of neural and behavioral phenotypes, which can be detected using the other computational methods described in this section, has great potential to reveal how interacting neural processes combine to generate and sustain behaviors associated with psychopathology, including the clinically relevant over-expression of anxiety. With this prospect in mind, the next section introduces relationships and distinctions between adaptive, pathological, and trait anxiety, to show where the application of computational models can have the greatest impact.

## State Anxiety, Trait Anxiety, and Anxiety Disorders

Anxiety disorders involve states of anxiety so frequent and intense they dominate and undermine a patient’s daily life, leading them to seek medical attention; however, states of anxiety serve an adaptive function if they are deployed appropriately, priming individuals to detect, and respond to danger. It follows that an intuitive biological hypothesis about anxiety disorders would be that they involve a primary malfunction in the brain mechanisms that regulate anxious states. Trait vulnerability to the development of anxiety disorders would then consist in less severe dysregulation of anxious states than that observed in a clinical anxiety disorder.

A conceptual problem with this view is that there are no objective rules that determine when an anxious state is appropriate; instead, anxious states are triggered on the basis of an individual’s lifelong learning about environmental dangers. The capacity to learn effectively about environmental dangers therefore constitutes an additional factor that determines whether or not an individual is likely to experience uncontrolled, obtrusive anxious states—but one that depends upon an extremely wide range of cognitive abilities, including (for example) memory, reinforcement learning, sensory discrimination, and attentional control. As a result, frequent symptoms of anxiety—as indexed by a high trait anxiety score—are unlikely to derive from a single causal factor that is consistent across individuals, even though these individuals share the common feature of experiencing frequent anxious states.

The first part of this section describes evidence for the adaptive function of state anxiety in situations where this function can be clearly defined. Subsequently, we introduce anxiety disorders as described in the DSM-5. Finally, we examine how a biologically mediated tendency to experience more anxious states may lead to the maladaptive responses observed in anxiety disorders.

### State Anxiety

The adaptive function of anxious states can be most clearly illustrated with reference to animal studies and related work on neurobiology. Ethological research, which addresses animal behavior in natural or “semi-natural” settings (86), has shown that rodents do not react to the potential presence of a natural predator (signaled by cat odor for example) in the same way they react to a definite and observable threat (a cat). In a classic experiment (86), cat odor prompted rats to engage in “risk assessment” and suppression of ongoing non-defensive behaviors whereas an actual cat caused rats to run into their burrows or freeze (86, 87). Separate test batteries were devised to measure these respective behavioral profiles and used to show that the former was much more susceptible to moderation by the administration of anxiolytic drugs than the latter (88).

Parallel research on the activity of the neurotransmitter serotonin suggested a neurobiological correlate of this behaviorally important distinction between responses to proximal and distal threats (42, 89). In an influential theory, Deakin and Graeff suggested that serotonergic signals from a region of the brainstem—the dorsal raphe nucleus—were involved in an “anticipatory anxiety system” (42) that simultaneously increased aversive processing in the amygdala and restrained activation of fight/flight behaviors, which is mediated by glutamatergic projections from the amygdala to the brainstem periaqueductal gray (90). When activated, this system would generate the risk assessment behaviors observed in ethological experiments, along with suppression of ongoing activities [see also Ref. (91)], allowing animals to make more sophisticated defensive responses and thereby increasing their chances of survival. Some predictions of this hypothesis about the neural substrates of threat processing have been corroborated in human neuroimaging experiments involving healthy controls (92) and patient groups (93). Taken together with animal work on risk assessment (94), it presents a consistent view of anxiety as a state that suppresses ongoing activity, keeps fight/flight responses at bay, and facilitates threat processing.

States of anxiety arising in response to an ambiguous threat can be studied in healthy human volunteers using threat of shock studies (95). In these studies, participants perform a behavioral task under two conditions: in one condition, they are told that they may experience a painful electric shock at any time; in the other, they are safe. Actual shocks are rare, but the possibility of a shock provokes a state of anxiety (96). Threat of shock has the overall effect of biasing participants away from task-directed (97, 98) and toward sensory processing (97, 99). It improves performance in threat detection tasks (100), but impairs performance in tasks involving emotional distractors (101). A recent neuroimaging study demonstrated that threat of shock is associated with increased neural activity correlated with aversive prediction error signals in healthy participants (50), consistent with the idea that threat learning is altered in states of anxiety. All these results support the view, derived from animal and pharmacological studies, that adaptive state anxiety facilitates threat detection and processing at the expense of other resource-demanding cognitive processes in the context of uncertain threat.

### Anxiety Disorders

Anxiety disorders, as described in the DSM-5, capture characteristic ways in which excessive or uncontrolled anticipation of uncertain threat can lead people to seek medical attention. The 11 anxiety disorders described in the DSM-5 are separation anxiety disorder; selective mutism; specific phobia; SAD; panic disorder; agoraphobia; GAD; substance/medication-induced anxiety disorder; anxiety disorder due to another medical condition; other specified anxiety disorder; and unspecified anxiety disorder. Diagnosis of an anxiety disorder typically follows persistence of symptoms for 6 months [(29), p. 189] and elimination of alternative explanations, including other psychiatric conditions.

The majority of anxiety disorders involve overwhelming and persistent states of anxiety experienced in relation to a particular context. Separation anxiety, for example, involves distress about potential separation from attachment figures [(29), p. 190]. Selective mutism involves failure to speak in particular social situations where doing so may cause embarrassment [(29), p. 195]. Specific phobia, social phobia, and agoraphobia are perhaps more familiar, and similarly involve fears and avoidance behaviors associated with relatively well-defined settings in which the nature of the threat remains somewhat diffuse.

Two further anxiety disorders are characterized by symptom profiles rather than responses to particular situations. In panic disorder, there is no circumscribed situation or set of objects that triggers panic attacks: panic attacks themselves, along with fear of further attacks, are the overriding features [(29), p. 208]. Notably, there is evidence that panic, in contrast to anxiety, involves different processes and brain structures, and in particular the periacqueductal gray matter (86, 89); here we are focusing on anxiety rather than panic, so uniquely panic-related processes and mechanisms will not be a focus. In GAD, anxiety and worry about a variety of issues, along with accompanying physical signs such as muscle tension and fatigue, are the characteristic symptoms [(29), p. 222].

All anxiety disorders are characterized by inappropriate or maladaptive manifestations of anxiety, which may conceivably emerge in any individual. Nevertheless, various biological factors render some individuals more vulnerable to the development of anxiety disorders [for a review considering psychological and neurobiological factors together, see Ref. (102)]. Precisely what these factors are, how they may best be characterized, and how they might interact with one another and the environment to make the development of clinical anxiety disorders more likely remains incompletely understood.

### Trait Anxiety

Threat-induced state anxiety and clinical anxiety disorders represent opposite extremes in terms of the adaptiveness of anxious states. Trait anxiety scores are commonly used as a proxy for proneness to experience maladaptive anxious states, but this self-report measure does not have a straightforward interpretation in terms of biology. In the case of the commonly used Spielberger State–Trait Anxiety Inventory Y2 score [STAI-Y2 (36)] it explicitly measures the frequency with which individuals report experiencing 20 separate characteristics of anxious states. Despite inevitable variability in the willingness to endorse particular characteristics (due, for example, to differing interpretations of specific words or phrases), the summed frequency score can be regarded as a good indication of how often someone has experienced anxious states over the course of their life [for a comparison with other self-report scales, see Ref. (103)].

Thus, although individuals with high levels of self-reported trait anxiety appear to be more prone to the development of some anxiety disorders (40), there is no reason to believe these people form a homogeneous group in terms of underlying biological risk factors (77, 104); what they share is a high self-reported frequency of experienced anxious states. Clearly, more frequently experienced anxious states may indicate exposure to a more anxiogenic environment, an increased biological predisposition to experience anxious states, or complex interactions between the two. Furthermore, biological risk factors are themselves likely to be diverse and different factors more or less significant in different environments.

**Figure 1:**
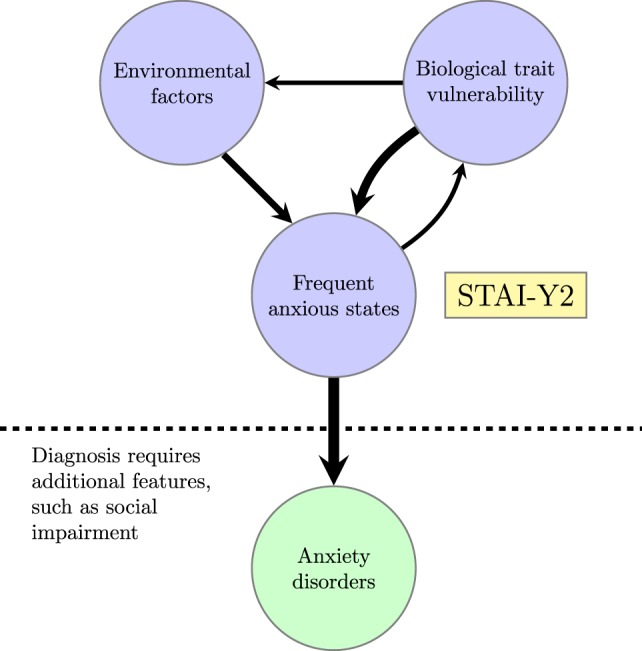
**Relationship between biological trait vulnerability, anxiolytic environment, self-report evidence of frequent anxious states, and diagnosed anxiety disorders; STAI-Y2 is the “trait” score on the Spielberger State-Trait Anxiety Inventory**.

This state of affairs is depicted in Figure 1. A lifelong tendency to experience frequent anxious states, as indexed by the STAI-Y2 score, increases the probability that someone will be diagnosed with an anxiety disorder. However, this tendency is itself the result of biological and environmental factors. The experience of frequent anxious states is assumed to feed back into biological factors through neuroplasticity (105) and epigenetic changes (106, 107), thereby opening a pathway for the effect of early stressful life events on biological vulnerability to the development of anxiety disorders (108–110). Biological vulnerabilities are assumed to affect environment factors directly by increasing attention to environmental threats, as described in the section, “Trait Anxiety: Targets for Computational Studies” [for other ways in which trait vulnerabilities might promote aversive experiences, see Ref. (102)].

Working from this conception of the relationship between trait anxiety, state anxiety, and anxiety disorders, the next section of this review draws on experimental evidence to decompose the trait vulnerability into key subcomponents. It focuses on interpreting behavioral and neuroscientific findings in computational terms in order to suggest appropriate targets for computational studies. The resulting conceptualization of the trait vulnerability is not meant to be definitive, but rather a starting point for future studies.

## Trait Anxiety: Targets for Computational Studies

Neuroscientific and psychological experiments have described various ways in which individuals high in trait anxiety differ from their less anxious counterparts. Some of these differences hint at altered underlying computational processes that could be captured using the modeling approaches described in the section on “Computational Background.” The current section focuses on four areas of interest: learning about threats, avoidance of danger, attention to threats, and frequency of experienced anxious states. The first three are highly susceptible to computational modeling; the fourth, which is indexed by self-reported trait anxiety, can be considered a consequence of them.

**Figure 2:**
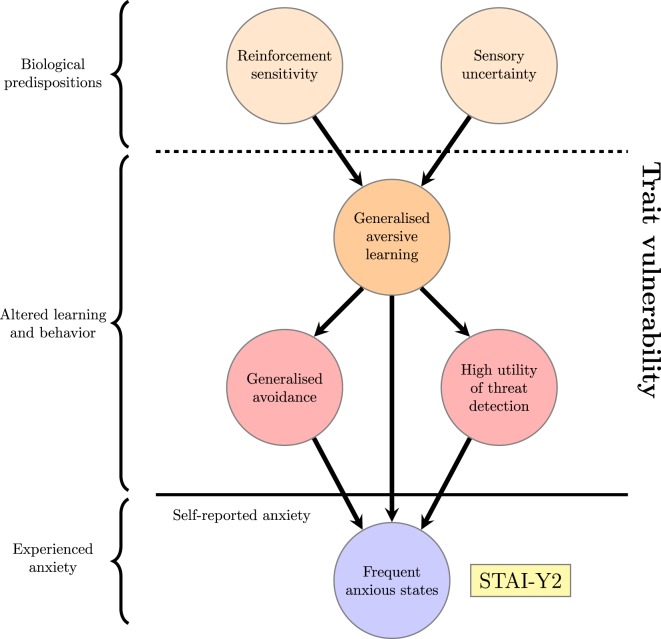
**Proposed factors underlying a high trait anxiety score; the trait vulnerability is decomposed into genetically mediated biological factors and resulting long-term alterations in learning**. This can be considered an unpacking of the “Biological trait vulnerability” node and accompanying thick arrow from the previous figure.

A schematic view of trait anxiety, where it is seen as resulting from these interacting computational processes, is shown in Figure 2. As described in the previous section, the term “anxiety” is used in experimental psychology and neuroscience to describe an unfocused response to diffuse, unpredictable threat [(35, 111, 112); though compare Ref. (113)]. It is distinct from responses to predictable and well-characterized threat, which are more correctly labeled as fear (114) and panic (89). The hypothesis illustrated in Figure 2 is that altered learning about threats, avoidance and attention alter the overall number of threats expected in the environment and render the distinction between threats and non-threats more ambiguous, leading to more frequent states of anxiety (115, 116).

Figure 2 thus illustrates how the self-report measure of trait anxiety is proposed to arise from underlying computational processes that will be further elucidated in the rest of this section. It should be noted that formulating this hypothesis is a different objective to describing state anxiety itself as a computational process—although that is a worthwhile and important goal [interested readers could see, for example, Ref. (117)]. Instead, it demonstrates how computational ideas might be combined with existing findings to provide insight into underlying factors that predispose some individuals to become anxious more frequently than others.

### Overgeneralization of Aversive Learning

Learning about aversive outcomes is often investigated using fear conditioning paradigms. In classical or Pavlovian conditioning (118, 119), a biologically significant “unconditioned stimulus” (US) is repeatedly presented following a predictive, initially neutral “conditioned stimulus” (CS). The US triggers suitable preparatory responses, such as salivation in the case of food; after a few repetitions these responses are evoked by the CS alone. In the case of an inescapable and inherently aversive US, such as electric shock, characteristic responses include fear-potentiated startle (120, 121) and increased galvanic skin response (122). Manipulation of factors influencing response transfer from US to CS (“acquisition” of the conditioned response) can be used to investigate complex underlying processes of associative learning [for a classic account, see Ref. (123)]. Since these learning processes can be described computationally using the theory of reinforcement learning [see Computational Background; also Ref. (124, 125)], behavioral measures in appropriately designed conditioning paradigms can be combined with reinforcement learning models to quantitatively assess them [see, for example, Ref. (126)].

A systematic review of fear conditioning in the anxiety disorders found that patients exhibited both stronger acquisition of conditioned responses and impaired differentiation between conditioned and non-conditioned stimuli [(127); see also Ref. (128)]. These effects led to generalization of conditioned fear responses (127)—for example, from a predictive CS to other perceptually similar stimuli (129). Generalization of fear responses was subsequently demonstrated among patients with panic disorder (130, 131) and GAD (132, 133). Similar effects have been found in healthy individuals with high levels of trait anxiety [(134, 135), but see Ref. (136)].

Importantly, generalization of fear conditioning to perceptually similar stimuli [such as circles with similar diameters, as in Ref. (129)] may only be the most straightforward example of wider trends in fear generalization prompted by sufficient levels of anxious arousal. Humans also reason symbolically, generalizing across abstract relations (137–139). This process may be particularly relevant for anxiety disorders whose symptoms concern situations that have not been directly experienced, but whose aversive nature is inferred from fear conditioning in related scenarios (140, 141). A small body of experimental work provides evidence for such “symbolic” fear generalization (142–145).

The core neural substrate of associative fear learning is a network of brain regions centered on the amygdala (146), whose neurobiology is understood in exquisite detail [for a recent review, see Ref. (147)]. It has been argued that individual differences involving associative learning dependent on the amygdala contribute to the development of anxious temperament and anxiety disorders both by driving specific behaviors and by biasing wider cognitive processing (148, 149). However, a recent review of the neurobiological basis of fear generalization (150) describes a variety of candidate or contributory mechanisms, including hippocampal pattern completion, cholinergic neuromodulation, and molecular factors within the amygdala. A recent brain imaging study investigating fear generalization (151) found that aversive and sensory information encoded in separate brain regions both contributed to the effect.

These early neurobiological results indicate that there may be several routes to fear generalization, with potentially significant consequences for its role in the development of anxiety disorders. For example, reinforcement sensitivity theories of personality posit that differences in reactivity to reinforcers underlie the long-term emergence of differences in temperament, such as anxiety (116). But if reactivity interacts with sensory discrimination to determine fear generalization (151), punishment sensitivity is only one of two factors that can lead a fear association to be generalized, alongside altered sensory processing. These factors would surely interact, but one or the other could be more important among different patient groups. In SAD, for example, fear generalization has not always been evident in laboratory tests that do not involve socially relevant stimuli (150) and has only been partially evident when such stimuli are involved (152). However, difficulties in sensory discrimination have been detected (153). Combined with real-life social situations involving specifically feared outcomes like embarrassment, sensory discrimination impairments could result in context-specific fear generalization.

Finally, although generalization of aversive associations has usually been investigated using fear conditioning paradigms, generalization could sometimes be more evident at the level of conscious reports than at the autonomic level (154), perhaps reflecting different neural substrates [i.e., hippocampal rather than amygdalar; see Ref. (155)] and consistent with cognitive “expectancy models” of fear acquisition (156, 157). Computationally, these differences could be detected using Bayesian models, which can be used to quantify the acquisition of expectations during perceptual learning (158) and readily combined with reinforcement learning approaches (69). Whether originating in reinforcement sensitivity or perceptual differences, generalization of aversive learning would always have the crucial result of increasing the number of fear-inducing stimuli in the environment, thereby raising the probability of initiating the anxious states described in the section on “State Anxiety.”

### Overgeneralization of Avoidance

The defining characteristic of an aversive stimulus, or punishment, is that it “is something an animal will work to escape or avoid” (159). Unlike pure associative learning, which underpins classical conditioning, successful avoidance requires implementation of an action. Among animals of a particular species, certain avoidance behaviors are instinctive punishment responses and can be transferred very easily from US to CS; others have to be learned based on their potential to facilitate escape [for a review, see Ref. (160)]. Though analogous to the learning of appetitive behaviors to obtain rewards, avoidance learning poses an additional theoretical challenge because reinforcement in this case depends on non-occurrence of an aversive event. This problem of so-called negative reinforcement can be solved by assuming that an aversive CS first acquires the power to generate fear by classical conditioning and that any action serving to remove the CS will be reinforced because it reduces fear (161, 162).

Anxiety may influence avoidance learning in two ways: by altering the process of classical conditioning used to build fear associations so that more stimuli generate fear and more actions (those that remove the fear stimuli) are negatively reinforced; or by shifting motivation toward avoidance rather than exploration so that avoidance is more of a priority than alternative actions. These effects would be mutually reinforcing rather than mutually exclusive. Evidence for the former effect comes from studies relating the generalized fear conditioning described in the section on “Overgeneralization of Aversive Learning” to subsequent patterns of avoidance behavior (163, 164). The latter, direct effect could be measured in behavioral paradigms orthogonalizing avoidance and exploration.

Working independently or in combination over a long period of time, these two mechanisms would have the overall effect of reducing an animal’s exploration of fear-associated regions of state space by making avoidance more likely (160). This would prevent the animal from learning that its fears were exaggerated. At the same time, negative reinforcement associated with apparently successful avoidance behaviors would further increase their likelihood of being repeated (165) and this process could become habitual in disorders involving compulsivity as well as anxiety [(166, 167); though see Ref. (168)].

Computationally, avoidance learning can be described by reinforcement learning models. One approach, based on the theory of negative reinforcement, is to use so-called actor–critic models (165, 169), which feature interconnected “actor” and “critic” modules that separately learn state and action values (45). The critic learns by a passive process akin to classical conditioning, which reduces the value of states that tend to predict an aversive outcome (equivalent to fear acquisition). The actor updates action values based on increases in state value obtained by leaving these low-valued states (reinforcement due to fear reduction). As a result, actions leading away from states predictive of punishment (i.e., avoidance behaviors) are reinforced [for a detailed explanation, see Ref. (165)]. Generalized avoidance could occur in such models by a variety of mechanisms, including oversensitive learning by the critic (so many states would take on negative values, leading to too many opportunities for negative reinforcement) and oversensitive learning in the actor (so even very small increases in state value could strongly reinforce an avoidance action). Intriguingly, these mechanisms could have differentiable neural substrates (47), perhaps offering a means of identifying characteristics of different subgroups of anxious individuals.

### Increased Expected Utility of Threat Detection

There is a substantial literature on altered attentional mechanisms among individuals with high levels of trait anxiety [see, for example, Ref. (170–174)]. These may include altered automatic threat evaluation [e.g., Ref. (170, 175)], difficulty disengaging from threat (176, 177), or impaired goal-directed relative to stimulus-driven attention [(178, 179); for a summary of all these theories and some others, see Ref. (174)]. Accordingly, attentional bias modification has been investigated as a treatment for anxiety (174, 180) and new directions continue to be explored [for example, enhancing attention toward positive stimuli (181)].

From a computational perspective, attentional biases can be interpreted as evidence of altered utility functions [for a related discussion, see Ref. (182)]. For example, difficulty disengaging from threats could reflect an increased expected utility of threat monitoring relative to alternative action choices. DDMs can be used to investigate value-based attentional mechanisms (183, 184) and could be applied to probe threat-related attentional biases among anxious subjects.

Attentional biases characterized by interference with working memory (178) may be linked to reduced recruitment of prefrontal regions for attentional control during conflict processing (185). Individuals exhibiting such biases can be seen as allocating increased expected utility to environmental scanning relative to alternative goal-directed processes. Like generalized avoidance, this could conceivably emerge as a long-term adaptation to a cognitive environment in which unpredictable dangers appear to be more abundant due to overgeneralization of aversive associations.

Behaviorally, this kind of adaptation may be expressed as increased agitation under conditions of uncertainty, since uncertainty further increases the need to monitor the environment. Learning under uncertainty can be explored using Bayesian models (186, 187), so it would be interesting to apply such models to examine whether anxious individuals expect more uncertainty within their environment. Combining such methods with measurements of attentional control could help to determine whether uncertainty increases the expected utility of information gathering over task-directed behavior and whether any such effects are enhanced among anxious individuals.

### More Frequent Anxious States

Identification and avoidance of a clear danger signal is a result of fear as long as it does not involve significant uncertainty (114). In a situation characterized by potential or ambiguous threat, it is not always clear which action might facilitate an escape to safety. The best option might not be to act immediately, but rather to inhibit action and wait for more information in the form of a change in the environment, recollection of an informative memory, or generation of a novel idea. It may even be appropriate to approach the threat (113). As described in the section on “State Anxiety,” evidence from ethological studies (86), pharmacology (88), and research on the neurobiology of the serotonin system (42, 89) suggests that such conflict situations promote a characteristic pattern of risk assessment and “behavioral inhibition” (91). Anxious states in humans are theorized to involve a similar process of conflict resolution (91) that has been equated with the subjective experience of “anxious rumination” [(188), p. 11], conceived as scanning for threats in one’s memory and imagination rather than the immediate environment.

Spatio-temporal proximity is often a crucial factor in determining whether a threat provokes a state of fear or anxiety (113), because more distal threats (for example, not having enough money upon one’s retirement) and the best means of avoiding them (in this case, various ways to save money) tend to be less clearly defined. It has previously been argued that trait anxiety consists in a reduced “defensive distance” for a given real distance to a threat (115) so that, for example, relatively far off risks would provoke risk assessment. This effect is typically ascribed to the interactions of neurotransmitter systems (116).

Defensive distance can also be understood at the level of learning processes. In an ecological setting, organisms do not face threats in isolation, but operate within an environment whose dangers they have learned about throughout their lives. As a result, their evaluation of the overall level of danger they face is based not just upon on their proximity to a single threat, but on what could be termed “background” or “mean” defensive distance, which would encompass their expected proximity to a threat given their overall learning about the environment. By increasing the number of stimuli that acquire fear associations, generalized aversive learning would reduce the mean defensive distance, effectively lowering the overall threshold at which specific threats would provoke a state of anxiety. A similar effect on defensive distance is proposed for increased expected utility of threat detection, because it would lead to more potentially threatening stimuli being detected.

As well as reducing defensive distance, generalized aversive learning could increase uncertainty about the nature of threats by compromising anxious individuals’ capacity to distinguish between safe and threatening circumstances. For example, generalizing fear (and thus avoidance) after a bad social experience with one person to others who were associated with them would increase uncertainty about where to find safe social interactions. Detection of more social threats (due to an increased expected utility of threat detection) would further increase such uncertainty.

Collectively these mechanisms are proposed to increase the frequency of anxious states by increasing the expected proximity of danger and uncertainty about its likely origin, even in relatively safe situations. Their collective influence is depicted in Figure 2, which can be considered an elaboration on the “Biological trait vulnerability” node from Figure 1 along with the thick arrow linking it to the self-report measure of “Frequent anxious states.” Their predicted relationships could be tested empirically using network methods [as for questionnaire and experimental data in Ref. (84)] if they were separately assessed within a stable cohort of experimental subjects. Such an approach has the distinct advantage of acknowledging that such biases reflect long-term trends in learning and behavior whose interrelationships cannot be directly assessed within a single experiment.

Crucially, state anxiety is characterized by processing and behavioral features likely to compound the effects of long-term trait vulnerabilities (100, 189). As a result, these factors would interact on a variety of timescales so that, for example, a bias toward reactive avoidance acquired over many years might lead to particularly jittery behavior when combined with a state-induced enhancement of aversive processing. Such state–trait interactions may underlie the incapacitating effect of anxious states on individuals with high levels of self-reported trait anxiety and constitute one important way in which frequent anxious states form positive feedback loops with trait vulnerability factors.

## Existing Computational Studies of Trait Anxiety

With a schematic view of computational processes underlying trait anxiety in place, this section describes existing computational studies of trait anxiety and considers how they might fit in to the framework illustrated in Figure 2. The account above indicates that trait anxiety involves a mixture of biologically driven learning biases and learned preferences shaped by these biases over time. Existing computational studies have approached trait anxiety both by analyzing mechanisms of aversive learning and detecting established preferences. As described above, these can be understood in relation to computational theories of Pavlovian conditioning (124), avoidance learning (165, 169), decision-making (53), and learning under uncertainty (65, 68).

### Altered Conditioning and Avoidance Learning

In a 2015 paper, Browning and colleagues assessed the performance of individuals with variable levels of trait anxiety in an avoidance learning task featuring two levels of environmental volatility. In the “low volatility” environment, participants could most effectively avoid an aversive stimulus by learning stable probabilistic associations between two possible action choices and the aversive outcome; in the “high volatility” environment, the action-outcome contingencies reversed at regular intervals. An ideal Bayesian learner adapts its rate of learning to account for changes in volatility, selecting a higher learning rate when contingencies appear to change (65). Browning et al. discovered that the degree of learning rate adaptation among experimental subjects was inversely correlated with trait anxiety, indicating that high trait anxious individuals were less adept at detecting changing contingencies. Furthermore, pupillometry revealed that low but not high trait anxious participants exhibited changes in pupil diameter correlated with volatility a few seconds after the outcome, suggesting that learning rate modulation was associated with a physiological process tracking volatility (71).

The “anxiety-related deficit in contingency learning” (71) detected in this study is consistent with findings that anxious individuals overgeneralize associative fear conditioning. This is because more general conditioning would reduce the distinction between fear responses to the two stimuli, interfering with statistical learning about the contingencies. Computationally, it manifests as “a deficit in the use of higher order statistics about the causal structure of adverse environments to guide decision-making” (71), and it seems to impair avoidance, since high trait anxious individuals were observed to make more mistakes on trials involving difficult choices (71). A possible neurological substrate of this effect is cholinergic modulation of sensory cortex by the nucleus basalis of Meynert, driven by increased activation of the central amygdala (150); reduced reactivity of the pupil to volatility could reflect a simultaneous inhibitory effect of heightened central amygdala activity on the Edinger–Westphal nucleus (190). These effects would be consistent with reduced modulatory activity in the amygdala-regulating ventromedial prefrontal cortex during fear conditioning (128).

Problems interpreting the causal structure of aversive experiences—derived from generalized fear conditioning—could further influence thoughts and actions by shifting motivation toward aversive responding and avoidance. This possibility can be investigated by presenting stimuli that have acquired fear associations through classical conditioning during instrumental tasks (191). Such stimuli simultaneously inhibit appetitive approach and promote withdrawal (191–193), indicating that tendencies toward approach and avoidance are subject to indirect contextual modulation by conditioned fear stimuli. By increasing the number of aversive Pavlovian influences within the environment, generalization of fear conditioning could theoretically potentiate both effects. The relative influence of inhibited reward-seeking and potentiated avoidance could depend in part upon interactions between serotonergic and dopaminergic neuromodulation because these neurotransmitters regulate action selection in different ways. For example, inhibition may be mediated by serotonin (194, 195) and response vigor by dopamine [(196); for a review, see Ref. (197)].

A pioneering computational account of serotonin’s role in the regulation of negative mood has described how variations in serotonin transporter metabolism that are found in the healthy adult population and associated with trait anxiety (198) could promote excessive inhibition of negatively valenced trains of thought (199). In this account, serotonin provides an inhibitory signal that reduces the probability an individual will explore thought processes likely to lead to affectively negative outcomes, analogous to increased avoidance (200). An under-active serotonin transporter increases the availability of serotonin, reducing exploration of states potentially leading to negative outcomes. Whenever an individual with this profile experienced a reduction in serotonergic neuromodulation [which could happen for various reasons—for an accessible description of factors affecting overall serotonin levels, see Ref. (201)], they would be exposed to the negative thought processes they had previously avoided. This would lead to a sudden increase in unexpected negative outcomes (199). Repeated experiences of this nature would make the world seem a more unpredictable and frightening place.

In relation to overgeneralized fear conditioning, it would be interesting to consider whether overproduction of serotonergic inhibitory signals by an excess of aversive conditioned stimuli could also increase avoidance. Dayan and Huys (199) describe a reinforcement environment in which rewards and punishments are symmetric and the level of serotonergic neuromodulation causes negatively valenced states to be avoided, leading to oversampling of positive states. Recent findings concerning the involvement of serotonin in reward and punishment signaling complicate this picture (202), but overgeneralized fear conditioning could lead the environment itself to appear weighted toward punishing outcomes by attributing negative valence to states that were actually benign. This would similarly restrict environmental sampling—this time limiting exploration of states that could lead to positive outcomes.

### Altered Threat Processing and Expectancies

In a 2010 paper, White and colleagues used a DDM (53, 54) to examine the relationship between threat processing and trait anxiety. In one experiment, participants performed a lexical decision task, which involves classifying strings of letters as words or non-words. Subjects chose between “word” and “non-word” for hundreds of letter strings, some of which were neutral and some threatening words. Their performance was initially assessed using accuracy and response time measures and higher trait anxiety found to be associated with non-significant trends toward faster and more accurate classification of threatening relative to non-threatening words. Subsequently, accuracy and response time measures were used to fit a DDM to the behavior of each participant and the fitted drift rate parameters (which express the speed at which a particle moves toward a decision boundary; see description in the section on “Computational Background”) were compared. Drift rates were significantly higher among individuals with high trait anxiety for threatening relative to non-threatening words.

This task addressed a seeming contradiction in the literature on trait anxiety: a cognitive model of anxiety (203) predicted generally heightened threat reactivity; but previous experimental work had failed to find an effect of trait anxiety on threat processing in experimental paradigms that did not involve input competition [(204, 205) for example; an example of a task that does involve input competition would be the emotional Stroop, described in Ref. (171)]. Since anxiety is associated with conflict resolution (91), the negative experimental findings could have indicated that it was input competition—and not threat processing itself—that was affected by trait anxiety. The results of White et al. (61) suggest that trait anxiety is associated with direct enhancement of threat processing even in the absence of input competition, consistent with theoretical predictions (203), but in contradiction to previous experimental findings. The non-significant trends in accuracy and response time indicate that—in this group of participants—such differences could have been overlooked without the use of a computational model.

As noted in the section on “State Anxiety, Trait Anxiety, and Anxiety Disorders,” transient states of anxiety in healthy participants also increase threat-related biases (95), demonstrating that such biases are subject to short-term modulation in the context of diffuse threat regardless of trait anxiety. A recent neuroimaging study addressed this issue using functional magnetic resonance imaging (fMRI) to detect activity-related changes in the blood-oxygenation-level dependent (BOLD) response that were correlated with aversive prediction error, alongside a threat of shock (96) manipulation of state anxiety to see how aversive learning changed under stress (50). Participants were required to predict whether particular cues would be followed by happy or fearful faces under probabilistic contingencies that ensured a steady stream of appetitive (happy) and aversive (fearful) prediction errors. The experiment was repeated in a “safe” condition and under threat of shock. When they were at risk of receiving an electric shock, participants reported higher levels of anxiety. In the same condition, aversive prediction error signals in the ventral striatum were significantly increased whereas appetitive prediction errors were unchanged. It would be interesting for future experiments to explore how these effects compare with threat biases attributable to trait anxiety such as those detected by White and colleagues.

As well as altered threat processing, trait anxiety is associated with altered threat expectations (157, 206, 207) and computational methods could be used to quantify and analyze the impact of these expectations in new and revealing ways. For example, a recent study used BDT (12, 69) to infer threat expectations from human behavior in a simulated approach-avoidance task (72). Participants had to choose when, if at all, to approach a reward while at various levels of risk from a “predator.” If they were caught by the predator they would lose all rewards previously collected in that stage of the task. A computational model based on BDT was designed to capture optimal behavior in the task under different prior expectations about reward-threat correlations: if threats were expected to correlate with reward onsets, participants would wait before approaching to collect a reward; otherwise, there would be no reason not to approach as soon as the reward appeared. Across four experiments, subjects tended to wait before approaching a reward, suggesting they had a prior expectation that threats and reward onsets were correlated. Since there were no such correlations in the task itself, the prior was taken to reflect a preexisting bias among experimental subjects (72).

Having quantified a bias toward behavioral inhibition in the face of approach-avoidance conflict (whose connection with anxiety is described in the section on “More Frequent Anxious States” above), the study goes on to examine how this bias might be altered among anxious individuals. Modulation of approach latency by threat probability and potential loss was increased among anxious participants, suggesting they were using an altered prior threat probability function (i.e., their threat expectations were different). The study’s achievement is thus threefold: it provides a quantitative measure of behavioral inhibition in the face of approach-avoidance conflict, links this to prior threat expectations and demonstrates that these expectations are altered among individuals with high levels of trait anxiety. In the future, this quantifiable example of anxiety-related behavioral inhibition could be adapted for fMRI experiments in order to explore brain activity likely to be abnormal among people with anxiety disorders as a result of heightened threat expectations.

## Outstanding Computational Questions

**Figure 3:**
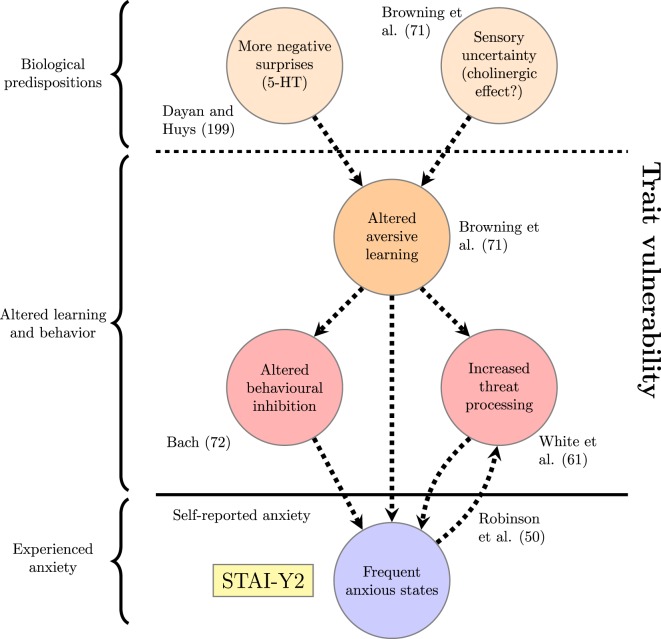
**Factors underlying trait vulnerability based on existing computational studies discussed in the section on “Existing Computational Studies of Trait Anxiety**.” The reference to Robinson et al. (50) justifies the arrow going back from “Frequent anxious states” to “Increased threat processing.” Dashed lines represent connections that do not have experimental support.

The examples above demonstrate that computational studies have produced a number of results characterizing altered aversive learning, threat processing and expectancies among individuals with high trait anxiety. But can they be related to the network of trait-defining processes derived from theoretical considerations in the section, “Trait Anxiety: Targets for Computational Studies”? For example, do they provide evidence of generalized aversive learning or avoidance, or of a connection between expected utility of threat detection and tendency to initiate anxious states? One possible mapping from the study results to the conceptual model is shown in Figure 3. This network is not meant to be definitive, but rather to illustrate the principle of considering particular results within a wider computational framework. The addition of an arrow from “Frequent anxious states” to “Increased threat processing” on the basis of Robinson et al. (50) demonstrates how a wider schematic view might be updated following a new result. Existing arrows in this version of the network are dashed to emphasize that the relationships they indicate are hypothetical and need to be investigated further. New arrows or nodes could be added based on additional studies, or the current nodes rearranged to express an alternative theoretical perspective.

One thing that is apparent both from this diagram and the preceding discussion is that computational studies can illuminate the processes underlying trait anxiety at different levels of detail. In particular, models can be devised with reference to functional outcomes (e.g., more frequent experienced anxiety), algorithmic processes of learning and action selection (e.g., generalization of fear conditioning), or specific biological mechanisms of interest (e.g., serotonergic signaling). These levels correspond to David Marr’s three levels of analysis—functional, algorithmic, and implementational—which are intended to capture “the different levels at which a device must be understood before one can be said to have understood it completely” (208). In doing so, they also capture different perspectives from which a computational process may be considered to have gone awry: that of what it is trying to achieve; that of the representations and algorithms it uses; and that of the mechanistic implementation of these algorithms. The rest of this section examines trait anxiety from these three perspectives to motivate new computational questions that could develop and refine the schematic view presented in Figure 2. An overview of relevant questions is provided in Table 1.

**Table 1:** **Starting points for future computational studies of trait anxiety**.

**Functional level**
How can we best model anxiety-like biases in conditioning and avoidance? When are these biases adaptive and when are they problematic? Can reduced attentional control be modeled as increased utility of threat detection? Can computational modeling provide a developmental account of anxiety disorders?

**Algorithmic level**
How does generalized fear conditioning influence decision-making and avoidance learning? Can these effects be understood using DDMs or reinforcement learning models? Can reinforcement schedules influence the utility of threat detection vs reward seeking? Can this be used to explore environmental influences on the development of anxiety disorders?

**Implementation level**
Do dopamine agonists facilitate active avoidance among anxious individuals? Can any such effects be linked to activity in the nucleus accumbens? Does increased cholinergic neuromodulation in sensory cortex generalize fear conditioning? How can this be modeled computationally?

**All levels**
How do trait-related biases interact with those observed during anxious states? Can network methods be applied to study interactions between computational processes?

At the most abstract level—that of functional outcomes—trait anxiety increases the resources devoted to threat detection, analysis, and avoidance relative to other activities, leading to more anxious states. Analyzing trait anxiety at this level means asking whether this goal is appropriate. In anxiety disorders it has clearly gone too far, which is why these conditions are categorized as disorders in the first place; in the case of trait anxiety the situation is less clear. Increased threat detection and avoidance confer clear evolutionary advantages on animals living in dangerous environments [for an intuitive example, see Ref. (209); memorably described in Ref. (210)] and it seems unlikely that there is an optimum baseline level equally suitable for all circumstances. Consistent with this idea, some arguments ascribe the current prevalence of anxiety disorders to evolutionary adaptations that only happen to be disadvantageous in the modern world (211).

In order to assess such arguments it would be necessary to consider under which circumstances biases associated with trait anxiety are beneficial and under which circumstances they are detrimental. As described by Bach (72), the establishment of normative models of anxiety, which emphasize its adaptive value in appropriate situations, is an important starting point from which to ask questions relevant to its dysfunction. It would be interesting to test a conceptual model whereby: (i) enhanced and overgeneralized fear conditioning (derived, for example, from high reinforcement sensitivity) increase the number of danger signals in the environment, resulting in more avoidance; (ii) this increases approach-avoidance conflict, so associated behaviors, such as environmental scanning, are used more readily; and (iii) over time, these behaviors are reinforced, so it becomes more likely they will be used in the future. In this way, generalized conditioning could result in a general tendency to prefer information-gathering behaviors at the expense of task-related activities—in other words, “anxiety looking for a threat to perceive” (91), which may tip into pathological hypervigilance in anxiety disorders.

Such developmental processes could be explored in computer simulations featuring agents with varying levels of reinforcement sensitivity. In any such simulations, the relationship between threat detection and the frequency of anxious states could undermine the adaptive value of increased threat detection for a biological system. Specifically, more frequent anxious states caused by greater uncertainty about the nature of threats in the environment and their proximity would themselves enhance threat-related biases, conceivably leading to feedback interactions between state anxiety and subcomponents of the trait vulnerability (i.e., the arrows between “Increased threat processing” and “Frequent anxious states” in Figure 3). Such interactions should be explored in future theoretical studies because they may determine the point at which an adaptive awareness of threats could become an unmanageable state of hypervigilance. In terms of experimental data, analysis of longitudinal data using network and experience sampling methods (81) could be used to determine whether individuals with high trait anxiety are more likely to display symptoms of anxious states after a negative experience, or perhaps continue displaying such symptoms for longer.

Some studies have already explored interactions between state and trait anxiety on shorter (i.e., within-experiment) timescales (212, 213) and computational accounts of the effects observed in these studies, or related effects, could also be developed. Reductions in attentional control due to stress, for example, could interact with over-general fear conditioning to severely disrupt concentration (the former effect increasing distractibility; the latter increasing the number of distractions). This could undermine anxious individuals’ ability to learn effectively about newly encountered threats, since reinforcement learning relies on attentional processes in complex environments (214). Alternatively, as described in the section, “Trait Anxiety: Targets for Computational Studies,” enhanced threat processing under stress could combine with a learned predisposition toward avoidance to result in erratic motor activity. Computational accounts of such processes could prove valuable in explaining how intrinsic vulnerabilities might promote systemic breakdown under specific circumstances.

Most existing computational work on trait anxiety has been carried out at the algorithmic level. In the experimental studies described in the section on “Existing Computational Studies of Trait Anxiety,” tasks are relatively well-defined, but individuals with varying levels of trait anxiety nevertheless differ in the way they avoid unpleasant outcomes (71), process threat-related information (61), or trade off approach and avoidance (72). Conditioning and avoidance biases will continue to be explored in experiments that compare average performance of groups with high and low trait anxiety, or seek correlations with a trait-anxiety regressor. Such experiments could investigate, for example, how generalized fear conditioning influences decision-making and avoidance [perhaps using reinforcement learning models to provide an algorithmic explanation for results such as those described in Ref. (163)]. In relation to attentional control deficits, modeling work could examine how particular reinforcement schedules, involving different levels of uncertainty, could enhance the expected utility of threat detection relative to reward seeking. Where severe and unpredictable punishments were highly likely, for example, it would presumably be advantageous to prioritize threat detection over other activities. In this case, a bias toward threat detection would follow from heightened threat expectations. Alternatively, modeling could be used to explore the question of how particular forms of threat detection leading to apparently successful punishment avoidance (for example, eye movements to check for something snake-like moving along the ground) could be reinforced. For example, could they be subject to the same process of negative reinforcement as avoidance actions themselves? In this case, threat detection would be conceived more as a habit, which was employed more frequently regardless of conscious threat expectations.

A further aim of future studies operating at the algorithmic level should be to assess relationships between different computational features of trait anxiety. Efforts in this area could benefit greatly from the application of network analysis (76) to measurable differences in computational learning processes. For example, individuals with high levels of trait anxiety struggle to adapt learning about aversive outcomes to changes in environmental volatility (71), react differently to approach-avoidance conflict (72) and process threatening information more rapidly (61), but would these effects be strongly correlated with one another if these experiments were carried out on the same subjects? And might one effect turn out to be more fundamental than the others? Answering such questions would help researchers develop new versions of the network depicted in Figure 2, providing greater insight into the ways algorithmic learning processes associated with trait anxiety interact over time to generate vulnerability to the development of anxiety and mood disorders.

At the implementational level, the potential role of cholinergic neuromodulation in trait vulnerability to anxiety has been discussed elsewhere (71, 150) and may be related to its hypothesized involvement in signaling expected uncertainty—that is, uncertainty arising from “known unreliability of predictive relationships” (215). Individual differences in the activity of acetylcholine could be a primary biological cause of increased sensory uncertainty about the relationships between percepts and aversive outcomes and this possibility could be investigated by building on previous modeling work describing the effects of cholinergic neuromodulation (216). Bayesian modeling approaches, with their capacity to represent various forms of uncertainty (186, 215), will be key to any such endeavors.

In relation to the activity of dopamine and serotonin, reinforcement learning models could be used to further examine variability of fMRI BOLD prediction error signals during avoidance learning, extending previous non-computational work (217). Combined with manipulations of serotonergic and dopaminergic neuromodulation [as in, for example, Ref. (196)], this approach could help elucidate the relative contributions of these neurotransmitter systems to expected alterations in avoidance learning associated with trait anxiety. For example, could high trait anxiety be associated with increased encoding of aversive value—and, if so, does this lead to increased encoding of reward value for avoidance? Could enhancement of dopaminergic neuromodulation more effectively facilitate active avoidance than active approach among individuals high in trait anxiety?

A final task for models operating at the implementational level will be to link findings about human trait anxiety to ongoing circuit-level research on the mechanisms underlying anxiety in animals [reviewed in Ref. (218, 219)]. This will allow for considerable elaboration of the “Biological predisposition” nodes in Figure 2. Since various biological mechanisms could conceivably lead to similar anxious phenotypes at the level of behavioral measurement or self-reported symptoms a full characterization of these possibilities will be necessary to provide the most effective treatments for anxiety disorders in the long run—and it is hard to imagine that this can be provided by experiments on human subjects alone.

## Conclusion

As a self-report measure of a long-term behavioral trend, trait anxiety is not the most obvious subject for computational study. It seems too intangible and loosely defined to be approachable with the precision tools of computational modeling. However, this review has sought to demonstrate that modeling can be a useful addition to the study of trait anxiety precisely because it forces scientists to be explicit about the details and relevance of their hypotheses. In particular, the computational techniques of reinforcement learning, decision modeling, Bayesian modeling, and network analysis can all be used to address pressing questions about the processes underlying trait vulnerability to the development of anxiety disorders—as well as the potential relationships between them.

Furthermore, two of the immediate challenges presented by trait anxiety—its basis in interactions between multiple cognitive processes and potentially considerable dependence on environmental factors—are likely to characterize any dispositional factor associated with mental illness. The study of trait anxiety provides an ideal opportunity to tackle these challenges with reference to a risk factor already characterized in exquisite detail over decades of intense neuroscientific study and theoretical analysis. As a result, it could turn out to be an effective testbed for the computational exploration of interactions between predisposing vulnerabilities and exposure to adverse environments, which contribute to the development of many psychiatric disorders.

## Author Contributions

JR was the main author of the paper. PS and JS contributed to writing the paper.

## Conflict of Interest Statement

The authors declare that the research was conducted in the absence of any commercial or financial relationships that could be construed as a potential conflict of interest.
